# Stereotactic radiofrequency lesioning of caudal zona incerta for parkinsonian tremor

**DOI:** 10.3171/2024.7.FOCVID2462

**Published:** 2024-10-01

**Authors:** Jitin Bajaj, Namrata Khandelwal, Anivesh Jain, Muddaiah N. Swamy, Yad R. Yadav

**Affiliations:** Departments of 1 Neurosurgery,; 2 Neurology, and; 3 Neuroanesthesia, Superspeciality Hospital, NSCB Medical College, Jabalpur, India; and; 4 Department of Neurosciences, Apex Hospital & Research Center, Jabalpur, India

**Keywords:** continuous tremor, deep brain stimulation, Parkinson disease, stereotactic technique

## Abstract

This video showcases stereotactic radiofrequency lesioning of the caudal zona incerta (CZi) for parkinsonian tremor in a 70-year-old patient. The preoperative evaluation, including imaging and frame placement, is detailed. The surgical procedure involves meticulous targeting and trajectory planning. Intraoperative stimulation is utilized for motor response assessment. Two temporary lesioning phases precede the final procedure at 75°C. The postoperative CT scan highlights the lesion site. Immediate tremor relief is observed postoperatively, with the effect persisting at the 1-month follow-up. Supporting readings underscore the efficacy and safety of CZi for tremor management.

The video can be found here: https://stream.cadmore.media/r10.3171/2024.7.FOCVID2462

## Transcript

In this video, we demonstrate stereotactic radiofrequency lesioning of caudal zona incerta for parkinsonian tremor.

### 0:27 Case Description.

A 70-year-old gentleman presented with Parkinson’s disease, primarily expressing left-sided upper-limb tremors that significantly impeded his daily activities. The severity of tremors, assessed using the UPDRS-III scoring for both rest and kinetic movements, was deemed moderate. Additionally, he exhibited mild tremors in other body parts, along with mild rigidity and bradykinesia. Despite therapeutic attempts with levodopa and carbidopa, trihexyphenidyl, and propranolol, the tremors proved resistant. Following a comprehensive discussion regarding the potential benefits and constraints of lesioning procedure, he consented to undergo stereotactic right-sided caudal zona incerta (CZi) radiofrequency thermocoagulation.

### 1:11 Preoperative Videos.

Herein, we can see the tremors at rest and during work.

### 1:30 Preoperative Imaging.

A preoperative magnetic resonance imaging (MRI) with thin sections and proton density fat saturation was conducted for improved visualization of the red and subthalamic nuclei.

### 1:41 Frame Placement and CT Scanning.

Administered under local anesthesia, the stereotactic frame (3DR Stereotactic System, Mahalasa Medical Technology) was meticulously aligned parallel to the orbitomeatal line, after which a thin-section computed tomography (CT) scan was performed.

### 1:55 Merging.

Both the MRI and CT scans were merged utilizing the inbuilt software.

### 2:18 Anterior Commissure–Posterior Commissure Marking.

The anterior commissure (AC) and posterior commissure (PC) were marked on MRI, and the imaging was reset according to the AC-PC line.

### 2:25 Target.

The red nucleus and subthalamic nucleus are marked with green dotted lines. The delineation of the caudal zona incerta was accomplished through direct visualization. At the section displaying the maximum dimensions of the red nucleus, three lines were demarcated: the first traversing the midpoint of the red nucleus, the second intersecting the lateral boundary of the red nucleus, and the third tangent to the medial edge of the subthalamic nucleus. On the line connecting the lateral border of the red nucleus and the medial border of the subthalamic nucleus, a point was marked at the two-third/one-third junction. The intended target within the CZi is precisely situated at or just posterior to this marked point.

### 3:03 Entry Point and Trajectory.

The entry point is marked at or anterior to the coronal suture approximately 2.5–3 cm off the midline. Care is taken to avoid ventricles, sulci, and vessels. If the trajectory involves the subthalamic nucleus, then the trajectory is medialized. In most cases, the trajectory of CZi and ventralis intermediate nucleus (VIM) can coincide with the CZi target just inferior to the VIM. The coordinates for the frame were noted.

### 3:32 Operating Room Procedure.

Thereafter, the patient was brought into the operating room, where the stereotactic arc was accurately positioned according to the predetermined coordinates. Following the planned approach, a burr hole was meticulously created at the designated entry point. Subsequently, a 2-mm-diameter radiofrequency electrode, featuring an exposed tip measuring 2 mm in length, was carefully inserted until reaching the target. The length of the electrode, set at 185 mm, adhered to the center of arc principle applicable to the utilized frame.

### 4:03 Stimulation and Lesioning.

During the procedure, the patient underwent intraoperative high-frequency stimulation at 100-Hz frequency, 1-msec pulse width, and 1.3–1.7 V to assess the motor response. Additionally, low-frequency stimulation was employed at 2-Hz frequency, 1-msec pulse width, and 1.3–3.5 V to establish the motor threshold. Remarkably, the patient experienced immediate relief from tremors upon high-frequency stimulation. Subsequently, two temporary lesioning phases were conducted: the first at 45°C for 30 seconds and the second at 55°C for another 30 seconds, aimed at evaluating potential adverse effects, such as paresis or speech changes. After a thorough assessment, the final lesioning procedure was performed at 75°C for 60 seconds.

### 4:57 Postoperative CT.

The postoperative CT scan delineates the location of the lesion.

### 5:03 Results.

The patient had immediate and complete relief from his tremors. Herein, we can compare the resolution of tremors both in rest and in kinetic conditions.

### 5:25 Follow-Up at 1 Month.

The effect persisted in the follow-up.

### 5:39 Suggested Readings.

There is class I evidence supporting the efficacy of deep brain stimulation in the caudal zona incerta for tremors.^1^ In contrast to VIM, which targets only the cerebellothalamic pathway, the CZi targets both the hyperdirect and cerebellothalamic pathway.^2^ The hyperdirect pathway plays a pivotal role in the therapeutic application of deep brain stimulation of the subthalamic nucleus for Parkinson’s disease. Moreover, CZi has demonstrated safety from a cognitive perspective in treating essential tremors.^3^ A comprehensive pooled analysis indicated the superiority of the posterior subthalamic area, i.e., CZi, over VIM in addressing essential tremors.^4^ While there is evidence for radiofrequency lesioning of CZi for posttraumatic tremor,^5^ herein we have illustrated its role in the case of parkinsonian tremor.

## Disclosures

The authors report no conflict of interest concerning the materials or methods used in this study or the findings specified in this publication.

## Author Contributions

Primary surgeon: Bajaj. Assistant surgeon: Swamy, Yadav. Editing and drafting the video and abstract: Bajaj, Jain, Yadav. Critically revising the work: all authors. Reviewed submitted version of the work: Bajaj, Khandelwal, Jain, Yadav. Approved the final version of the work on behalf of all authors: Bajaj. Supervision: Jain, Swamy.

## Supplemental Information

### Patient Informed Consent

The necessary patient informed consent was obtained in this study.
